# Small Defects Detection of Galvanized Strip Steel via Schatten-*p* Norm-Based Low-Rank Tensor Decomposition

**DOI:** 10.3390/s25082606

**Published:** 2025-04-20

**Authors:** Shiyang Zhou, Xuguo Yan, Huaiguang Liu, Caiyun Gong

**Affiliations:** 1 Key Laboratory of Metallurgical Equipment and Control Technology, Ministry of Education, Wuhan University of Science and Technology, Wuhan 430081, China; zhoushiyang@wust.edu.cn (S.Z.); yanxuguo@wust.edu.cn (X.Y.); liuhuaiguang@wust.edu.cn (H.L.); 2 Hubei Key Laboratory of Mechanical Transmission and Manufacturing Engineering, Wuhan University of Science and Technology, Wuhan 430081, China; 3 State Key Laboratory of Intelligent Manufacturing Equipment and Technology, Huazhong University of Science and Technology, Wuhan 430074, China

**Keywords:** galvanized strip steel, small target detection, Schatten *p*-norm, tensor decomposition

## Abstract

**Highlights:**

**What are the main findings?**

- Compared with the deep learning method for surface defect detection, the proposed SLRTD-based detection method is simpler and more effective in a galvanized strip steel production line.

**What is the implication of the main finding?**

- The proposed SLRTD-based detection method of surface defect can be applied for other industrial products, such as glass, fabric, LCD, and AMOLED.

**Abstract:**

Accurate and efficient white-spot defects detection for the surface of galvanized strip steel is one of the most important guarantees for the quality of steel production. It is a fundamental but “hard” small target detection problem due to its small pixel occupation in low-contrast images. By fully exploiting the low-rank and sparse prior information of a surface defect image, a Schatten-*p* norm-based low-rank tensor decomposition (SLRTD) method is proposed to decompose the defect image into low-rank background, sparse defect, and random noise. Firstly, the original defect images are transformed into a new patch-based tensor mode through data reconstruction for mining valuable information of the defect image. Then, considering the over-shrinkage problem in the low-rank component estimation caused by a vanilla nuclear norm and a weighted nuclear norm, a nonlinear reweighting strategy based on a Schatten p-norm is incorporated to improve the decomposition performance. Finally, a solution framework is proposed via a well-designed alternating direction method of multipliers to obtain the white-spot defect target image by a simple segmenting algorithm. The white-spot defect dataset from a real-world galvanized strip steel production line is constructed, and the experimental results demonstrate that the proposed SLRTD method outperforms existing state-of-the-art methods qualitatively and quantitatively.

## 1. Introduction

Galvanized strip steel is widely used in automobile manufacturing, household electrical appliances, and other daily-use products; surface defects might lead to some product quality issues for both in-progress and downstream products. The white-spot defects mainly caused by random zinc dross and ash in the hot dip or electro galvanizing process are considered the most serious threat to the steel surface quality due to their high concurrence and typical periodicity. This defect can be detected and recorded by the surface defect inspection based on machine vision, and then the quality issues with strip steel will be controlled at the early stage [1,2]. In fact, industrial production images frequently contain some prior information; for example, if a defect rarely appears, the vast majority of images show no defects. The white-spot defects are always ultra-tiny in size, occupying less than 5% of the whole raw image. The inherent issues of tiny size and low-contrast of white-spot defects under complex clutters and heavy noise constitute the significant obstacle in the detection and segmentation of the defect target: (a) they are sensitive to tiny defects because of their extreme small sizes, and (b) they cannot accurately locate the random appeared defects whose semantic relationship with the background context is weak.

In industrial machine vision, small or tiny object detection has always been a significantly studied issue, especially in industrial applications [3,4,5,6,7]. The research can be classified into three categories: traditional filtering-based methods, sparse and low-rank representation-based methods, and data-driven methods [8]. Filtering-based methods typically design relevant filtering operators in either grayscale or derivative spaces to suppress background noise and enhance targets. While these methods offer a certain level of real-time performance, they often require manual design of operators tailored to specific scenes when facing complex backgrounds, which limits their generalization ability and adaptability. Unlike the methods above that rely on manually designed features based on data priors, data-driven approaches utilize convolutional neural networks (CNNs) for deep feature extraction automatically [9,10]. Although these methods perform well and can extract deeper information, they depend heavily on image samples, which affects their real-time applicability. Due to the size limitations of small targets, which typically consist of only a few pixels, there is a risk of losing critical information during target detection, which may be ineffective or even degrade detection performance. In contrast, some traditional methods offer strong interpretability and maintain a certain level of robustness when faced with complex scenarios, maintaining certain advantages.

**Figure 1:**
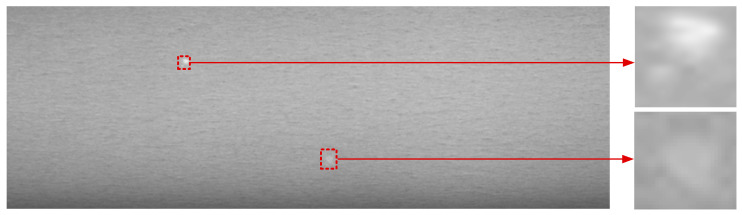
White-spot defects on the surface of galvanized steel sheet: the rightmost is the proportion of defective pixels in the whole image.

In recent years, tensor decomposition-based sparse and low-rank representation methods have achieved remarkable success, especially in infrared or remote sensing small target detection [11,12,13,14,15]. These methods leverage the low-rank nature of the infrared background and the sparse characteristics of small infrared targets; the background is modeled as a low-rank component, while the infrared targets are typically modeled as sparse components. The tensor model effectively preserves spatial structures and utilizes temporal information across multiple frames, which helps achieve more accurate target detection. These methods can combine spatial and temporal local information and iteratively optimize to approach the optimal detection results, significantly improving robustness to detect small targets in complex scenes. As the defect is random in the production line of strip steel, different image frames are irrelated, and the spatial and temporal information between different image frames are lost. As single-frame information is limited, these methods often miss targets or retain excessive false alarms when dealing with edge overlapping or complex backgrounds, thus failing to provide optimal decomposition guidance without temporal information across frames [16]. As shown in Figure 1, the white-spot defect of galvanized steel sheet is random and keeps changing all the time; small or tiny white-spot defects among 4096 × 2048 raw images are still easily ignored due to insufficient appearance information. At the same time, the highly varied nature of the background image and small target characteristics make the detection process extremely difficult.

Motivated by the above discussions, we establish the adaptive tensor model to accurately detect white-spot defects on galvanized steel sheet. The main contributions of this article are outlined as follows:

1. We propose an SLRTD method by digging out inter-patch correlation-ships of surface defect images of galvanized strip steel. The separated defect foreground target information with sparse outliers is embedded in the background of low-rank representation.
2. To achieve an accurate estimation of non-defect background rank, we incorporate weighted Schatten p-norm regularization for the background component, allowing for better noise removal while preserving edges, ultimately leading to improved detection results. Concurrently, a nonlinear reweighting strategy and tensor singular value decomposition (t-SVD) are adopted to help the model more delicately balance the low-rank and sparse components throughout the iterative process, which elevates the separation accuracy between the defect target and non-defect background.
3. According to the alternating direction method of multipliers (ADMM), we solve the sparse and low-rank decomposition problem. Experiment results demonstrate the feasibility and effectiveness of the proposed SLRTD method.

The remainder of this paper is structured as follows: Section 2 provides a concise overview of related work. Section 3 presents a comprehensive introduction to the architecture and components of the proposed SLRTD approach. Section 4 demonstrates numerical comparisons and module analysis. Finally, Section 5 concludes the paper. In our paper, tensors, matrices, and vectors are denoted using calligraphic letters, uppercase, and lowercase italics.

## 2. Related Works

In this section, we provide a brief overview of existing defect detection methods based on filtering, data-driven, and tensor decomposition methods.

### 2.1. Filtering-Based Methods

Generally, filter-based methods design relevant filtering operators in grayscale or derivative spaces to suppress background clutter and enhance the target. These methods can be further classified into the threshold method (e.g., Otsu, cross-entropy), morphological method (e.g., morphological operations, template matching), and spectral method (e.g., Fourier transform, Gabor transform, wavelet transform). However, the threshold and morphological methods often need manual adjustments to find the defect regions, especially when the distributions of scene intensity and texture are complicated. The images are transformed to frequency–domain in the spectral method, which often miss some small defects targets. These methods show poor adaptability and robustness [17].

### 2.2. Data-Driven-Based Methods

During the past few years, tremendous efforts have been devoted to small defect target detection based on deep learning [18]. Zhang et al. [19] constructed a novel feature enhancement network to improve the performance of small object detection. Hou et al. [20] proposed a contextual information and spatial attention that is based on a network for detecting small defects in the manufacturing industry. Yang et al. [21] introduced an RSADUnet model for metal surface tiny defect inspection. However, the satisfactory performance of these methods relies heavily on expensive labeled images, large numbers of parameters, and computational complexity to obtain strong feature awareness. Additionally, they are always used for natural or medical scenes where the shape of the specific object does not change much. Thus, these methods may not be suitable for steel surface defect detection in which the defect is random, especially for small defects with varied shapes and sizes. At the same time, a small defect occupies small pixels and lacks obvious texture and structure characteristics, thus it is difficult to provide reliable defect features.

### 2.3. Tensor Decomposition-Based Methods

In contrast, the tensor decomposition-based sparse and low-rank model always transforms the detection problem into an iterative optimization task by exploring new representations of the target and background features. Each patch is directly used as the obverse side slice to construct tensor data, which can guarantee the local features of the defect image can be reserved and is conductive to withdrawing the prior information. Many methods proposed different types of regularization to constrain low-rank and sparse attributes. The reweighted infrared patch-tensor (RIPT) model proposed by Dai et al. [22] utilizes the target sparse prior and background nonlocal self-correlation prior, and the local structure weight is designed for the target tensor term and serves as an edge indicator in the weighted model. However, RIPT still has limitations because the nuclear norm cannot accurately estimate the background. To alleviate this issue, inspired by t-SVD and a non-convex approximation of rank based on the tensor nuclear norm, Lu et al. [23] exploited the tensor robust principal component (TRPCA) method, which regularizes all singular values of tensor data equally and shrinks all singular values with the same parameter. Zhang et al. [24] introduced a novel nonconvex low-rank constraint, the partial sum of the tensor nuclear norm (PSTNN), combined with a weighted l1 norm to effectively suppress the background while effectively preventing target over-shrinkage. Gao et al. [25] developed an enhanced TRPCA (ETRPCA) model by the weighted tensor Schatten p-norm minimization, which makes the large singular values shrink less in the tensor nuclear norm minimization. The Schatten p-norm can better approximate the NP-Hard problem, optimize the recovery of its convex relaxation, and will not over-shrink the low-rank components of the data. Chen et al. [26] introduced logarithmic norm regularization as the nonconvex surrogate of matrix and tensor rank, which achieves more accurate low-rank approximation and high computational efficiency.

As it does not consider the influence from noise and clutter, the target detection effect will be reduced greatly in the face of the defect image in a more complex background. Luo et al. [27] utilized the sparse components of small targets along with a background dictionary, employing the fully connected tensor nuclear norm for low-rank estimation. Wang et al. [28] built a unique regularization term as a tensor-correlated total variation, which essentially encodes both low-rank and sparse priors of a tensor simultaneously. Geng et al. [29] developed a nonconvex and nonlocal TRPCA (NN-TRPCA) model based on the tensor adjustable logarithmic norm, which adaptively shrinks small singular values more and shrinks large singular values less. Huang et al. [30] introduced a two-stage feature complementary improved tensor low-rank sparse decomposition method, which is divided into two stages: tensor initialization and tensor decomposition, effectively integrating local and nonlocal features. Based on above analysis, the detection capability of these methods is dependent on the contrast between the target and the background. When there is strong contrast, these methods yield good detection results; otherwise, they tend to have a high false-positive rate. Moreover, these methods depend on the measurement of background rank in terms of accuracy, and obviously reduce performance and are time-consuming in the face of dim targets with complex backgrounds.

## 3. Methodology

**Figure 2:**
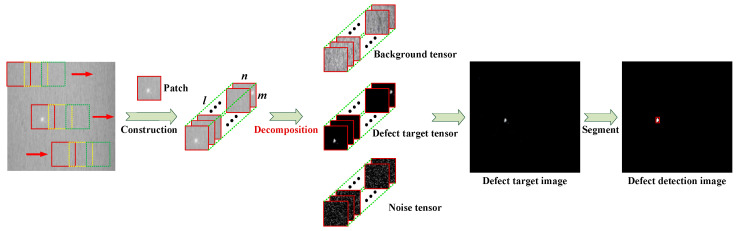
The flowchart of the proposed SLRTD method for surface defect detection.

The overall framework of the proposed SLRTD method is shown in Figure 2, which consists of construction of tensor model for defect image, model solution, and model analysis.

### 3.1. Construction of Tensor Model for Defect Image

Tensor can be expanded into matrix along *n*-modes and its *i*-th frontal slice are denoted as $X^{\left(i\right)}$. We denote $\bar{X}\in {\mathbb{R}}^{n_{1}\times n_{2}\times n_{3}}$ as the result of Discrete Fourier Transformation (DFT) along its third dimension by using $\bar{X}$, that is, $\bar{X}=\mathrm{fft}\left(X,\left[\;\right],3\right)$. The inverse operator computes X from $\bar{X}$, that is, $X=\mathrm{fft}\left(\bar{X},\left[\;\right],3\right)$. The tensor nuclear norm is defined as $\|X{\|}_{*}=\sum_{i=1}^{r}S\left(i,i,j\right)=\frac{1}{n_{3}}\sum_{i=1}^{r}\sum_{j=1}^{n_{3}}\bar{S}\left(i,i,j\right)$, where $r={\mathrm{rank}}_{t}\left(X\right)$ and $\bar{S}\left(i,i,1\right)$ are the entries on the diagonal of the first slice of $\bar{S}$, which has a decreasing order property. And we denote the l0 norm, the l1 norm, and the Frobenius norm as $\|X{\|}_{0}$, $\|X{\|}_{1}$, $\|X{\|}_{F}$, respectively.

The defect image is mainly composed of defect target, background, and noise, which can be expressed as follows:

(1) $$f_{D}=f_{T}+f_{B}+f_{N}$$

where $f_{D}$, $f_{T}$, $f_{B}$, and $f_{N}$ represent the input of original defect image, defect image, background image, and noise defect, respectively.

The original defect image I is two-dimensional data, which can be converted to tensor data. Assume original defect image I and the tensor structure obtained after data reconstruction is D. The image I will be browsed by m×n sliding window from left to right and from top to bottom; the total sliding time is l; the small patch obtained each time as the frontal slice of $D\in {\mathbb{R}}^{m\times n\times l}$ is shown in Figure 2. The tensor model of defect image can be constructed as follows:

(2) D=T+B+N

where D, T, B, and N represent defect image patch-tensor, defect target patch-tensor, background patch-tensor, and random noise patch-tensor, respectively.

Defect target patch-tensor T:

For defect image from the strip steel production line, defects with fewer pixels with respect to the whole image, it is sparse compared to most of the normal background regions. Thus, the corresponding defect target patch-tensor is an extremely sparse tensor, which can be depicted as follows:

(3) $${\left\|T\right\|}_{0}\leq d$$

where d is an integer that is related with defect target characteristics of number and size.

**Figure 3:**
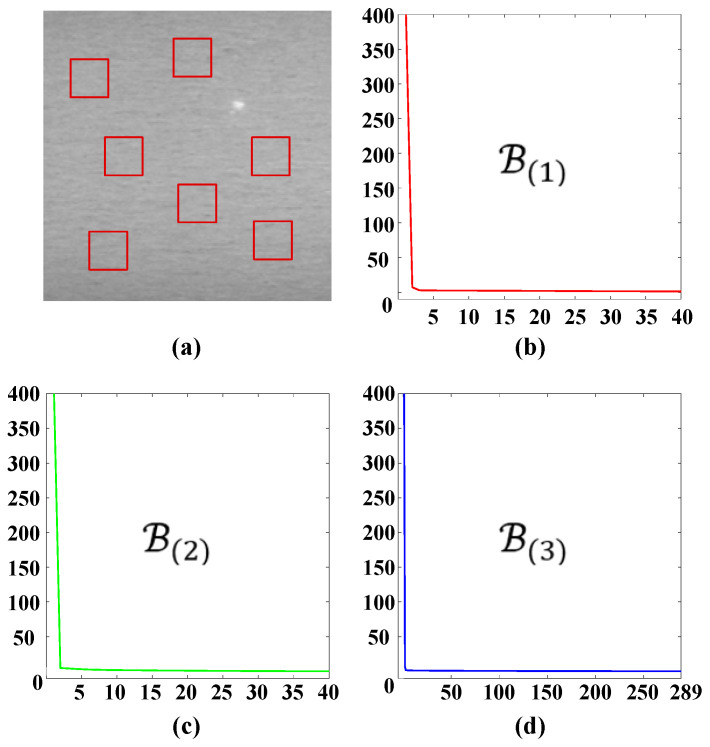
Illustration of the nonlocal similarity and the low-rank property of background patch-tensors: (**a**) one representative defect image; (**b**–**d**) corresponding singular values distributions curve of the mode-1, mode-2, and mode-3 unfolding matrices of background patch-tensors.

Background patch-tensor B:

As illustrated in Figure 1, the gray-value of normal regions is almost uniform, which means that the local patches are highly correlated with each other. Actually, the mode-1, mode-2, and mode-3 unfolding matrices of the background patch-tensor are also low-rank. In Figure 3b–d, the horizontal axes denote the number of singular values; the vertical axes denote the singular values. Assuming the defect image is 200 × 200, the patch is 40 × 40 and the step size is 10, the patch-tensor $D\in {\mathbb{R}}^{40\times 40\times 289}$, the size of mode-1, mode-2, and mode-3 unfolding matrices are 40 × 11,560, 40 × 11,560, 289 × 1600, respectively. From Figure 3, it can be observed that the singular values of three mode-unfolding matrices all exhibit a sharp decreasing trend and decline into approximate zero rapidly, with only a small fraction being significantly greater than zero. It indicates that the background patch-tensor B is correlated along each mode direction and further reflects its inherent low-rank structure within the subspaces corresponding to each mode [31,32]. Based on this property, we can consider the background patch-tensor B as a low-rank tensor, and their unfolding matrices are also all low-rank defined as follows:

(4) $$\left\{\begin{array}{l} \mathrm{rank}\left(B_{\left(1\right)}\right)\leq r_{1}\\ \mathrm{rank}\left(B_{\left(2\right)}\right)\leq r_{2}\\ \mathrm{rank}\left(B_{\left(3\right)}\right)\leq r_{3} \end{array}\right.$$

where $B_{\left(1\right)}$, $B_{\left(2\right)}$, and $B_{\left(3\right)}$ denote model-1, model-2, and model-3 unfolding matrices of B, respectively; $r_{1}$, $r_{2}$, and $r_{3}$ denote the background complexity.

Noise patch-tensor N:

The noise is usually modeled as additive white Gaussian noise, and it satisfies ${\left\|N\right\|}_{F}^{2}\leq \delta$, where δ>0 denotes the Gaussian noise level.

Based on above analysis, the small white-spot defects detection on galvanized strip steel based on tensor decomposition can be formulated as follows:

(5) $$\begin{array}{l} \underset{T,B,N}{\min}\left(\mathrm{rank}\left(B\right)+\lambda {\left\|T\right\|}_{0}+\eta {\left\|N\right\|}_{F}^{2}\right)\\ s.t.\;D=B+T+N \end{array}$$

where $\mathrm{rank}\left(\cdot \right)$ denotes matrix rank and ${\left\|\cdot \right\|}_{0}$ denotes $l_{0}$ norm, ${\left\|\cdot \right\|}_{F}^{2}$ denotes F norm, λ and η denote regularization parameters to balance the low-rank and sparse characteristics.

A reweighted strategy is always adopted to solve low-rank and sparse tensor decomposition. The principle of weighted Schatten p-norm minimization (WSNM) is to assign different weights to different singular values [33,34], which can recover the low-rank component and separate target from background more accurately by adjusting p. Therefore, we incorporate the WSNM to improve the detection accuracy of defect target. It can be defined as follows:

(6) $${\left\|B\right\|}_{W}^{p}=\frac{1}{n_{3}}\overset{r}{\underset{i=1}{\sum }}\overset{n_{3}}{\underset{j=1}{\sum }}{\left(W\left(i,i,j\right){\left(\bar{S}\left(i,i,j\right)\right)}^{p}\right)}^{\frac{1}{p}}$$

where $n_{3}$ is the number of patches, $W\left(i,i,j\right)=\frac{\xi \sqrt{n_{1}n_{2}}}{\bar{S}\left(i,i,j\right)+E_{B}}$ denotes a weight tensor, which is defined in Algorithm 1; $\bar{S}\left(:,:,j\right)$ represents the singular values of $B\left(:,:,j\right)$, ξ is a parameter, $E_{B}$ is a positive constant.
**Algorithm 1:** Solving Equation (11)**Input: **$X\in {\mathbb{R}}^{n_{1}\times n_{2}\times n_{3}}$,
p **Output: **$F_{\lambda }\left(X\right)$ **step 1:** Conduct FFT operation: $\bar{X}=\mathrm{fft}\left(X,\left[\;\right],3\right)$ **step 2:** Conduct SVD operation on each frontal slice ${\bar{X}}^{\left(i\right)}$ of $\bar{X}$ **      for**
$i=1,2,\ldots ,\lceil \frac{n_{3}+1}{2}\rceil$ **do**        $\left[U,\Sigma ,V\right]=\mathrm{svd}\left({\bar{X}}^{\left(i\right)}\right)$, $\Sigma =\mathrm{diag}\left(\sigma_{1},\ldots ,\sigma_{r}\right)$        Compute $w=\left[w_{1},w_{2},\ldots ,w_{r}\right]$          **for**
j=1,2,…,r **do**              $\delta_{i}=\mathrm{GST}\left(\sigma_{i},\lambda w_{i},p\right)$          end for        $\Sigma =\mathrm{diag}\left(\delta_{1},\ldots ,\delta_{r}\right)$, ${\bar{W}}^{\left(i\right)}=U\Sigma V^{T}$      **end for**      **for**
$i=\lceil \frac{n_{3}+1}{2}\rceil +1,\ldots ,n_{3}$ **do**           ${\bar{W}}^{\left(i\right)}=\mathrm{conj}\left({\bar{W}}^{\left(n_{3}-i+2\right)}\right)$      **end for** **step 3:** Compute $F\left(X\right)=\mathrm{ifft}\left(\bar{W},\left[\;\right],3\right)$

Additionally, $l_{0}$ norm is replaced by $l_{2,1}$ norm for solving the model. Hence, Equation (5) can be transformed as follows:

(7) $$\begin{array}{l} \underset{T,B,N}{\min}\left({\left\|B\right\|}_{W}^{p}+\lambda {\left\|T\right\|}_{2,1}+\eta {\left\|N\right\|}_{F}^{2}\right)\\ s.t.\;D=B+T+N \end{array}$$

### 3.2. Model Solution

In order to solve Equation (7), the ADMM are selected to solve the separable convex optimization problem. The Lagrange function can be constructed as shown in Equation (8), which can be converted to Equation (9), where, $Y\in {\mathbb{R}}^{r\times r\times n_{3}}$, μ and 〈·〉 denote the Lagrangian multiplier tensor, inner product among tensors, respectively.

(8) $$\begin{array}{lll} O\left(B,T,N,Y,\mu \right) & = & {\left\|B\right\|}_{W}^{p}+\lambda {\left\|T\right\|}_{2,1}+\eta {\left\|N\right\|}_{F}^{2}+\frac{\mu }{2}\|D-B-T-N{\|}_{F}^{2}+\\ & & \langle Y,\;D-B-T-N\rangle \end{array}$$

(9) $$O\left(B,T,N,Y,\mu \right)={\left\|B\right\|}_{W}^{p}+\lambda {\left\|T\right\|}_{2,1}+\eta {\left\|N\right\|}_{F}^{2}+\frac{\mu }{2}\|D-B-T-N+{\frac{Y}{\mu }\|}_{F}^{2}$$

Based on ADMM, O can be broken into several sub-problems for iterative update:

(i) Fix the rest of the variables and update of B, which is defined by Equation (10):

(10) $$B^{k+1}\begin{array}{c} =\arg\underset{B}{\min}\left(\frac{1}{2}\|D-T^{k}-N^{k}+\frac{Y^{k}}{\mu^{k}}-B^{k}{\|}_{F}^{2}+\frac{1}{\mu^{k}}{\left\|B\right\|}_{W}^{p}\right) \end{array}$$

It can be solved by Generalized Soft-Thresholding (GST) and tensor singular value thresholding (t-SVT) [35] as follows:

(11) $$B^{k+1}\begin{array}{c} =F_{\frac{1}{\mu^{k}}}\left(D-T^{k}-N^{k}+\frac{Y^{k}}{\mu^{k}}\right) \end{array}$$

where $F\left(\cdot \right)$ denotes solving WSNM-tensor problem, and the detailed process is provided in Algorithm 1. ${\left(\cdot \right)}^{k}$ represents the value of variables in *k*-th iteration.

(ii) Fix the rest of the variables and update of T, which is confirmed by Equation (12):

(12) $$T^{k+1}\begin{array}{c} =\arg\underset{T}{\min}\left(\lambda {\left\|T\right\|}_{2,1}+\frac{\mu^{k}}{2}\|D-B^{k+1}-N^{k}+\frac{Y^{k}}{\mu^{k}}-T{\|}_{F}^{2}\right) \end{array}$$

As $l_{2,1}$-norm of T is defined as the sum of $l_{2}$-norm of each mode-2 fiber, we matricize each tensor along the 2nd mode, so $\|T^{k+1}{\|}_{2,1}=\|T_{\left(2\right)}^{k+1}{\|}_{2,1}$. It can be transformed into the matrix form with Equation (13):

(13) $$T_{\left(2\right)}^{k+1}\begin{array}{c} =\arg\underset{T_{\left(2\right)}}{\min}\left(\lambda \|T_{\left(2\right)}{\|}_{2,1}+\frac{\mu^{k}}{2}\|D_{\left(2\right)}-B_{\left(2\right)}^{k+1}-N_{\left(2\right)}^{k}+\frac{Y_{\left(2\right)}^{k}}{\mu^{k}}-T_{\left(2\right)}{\|}_{F}^{2}\right) \end{array}$$

Let $X=D_{\left(2\right)}-B_{\left(2\right)}^{k+1}-N_{\left(2\right)}^{k}+\frac{Y_{\left(2\right)}^{k}}{\mu^{k}}$, so

(14) $$T_{\left(2\right)}^{k+1}\begin{array}{c} =\arg\underset{T_{\left(2\right)}}{\min}\left(\lambda \|T_{\left(2\right)}{\|}_{2,1}+\frac{\mu^{k}}{2}\|X-T_{\left(2\right)}{\|}_{F}^{2}\right) \end{array}$$

According to [36], it has the following close-form solution with Equation (15):

(15) $$T_{\left(2\right)}^{k+1}\left(:,j\right)\begin{array}{c} =\left\{\begin{array}{l} \frac{\|X{\left(:,j\right)\|}_{2}-\frac{\lambda }{\mu^{\left(k\right)}}}{\|X{\left(:,j\right)\|}_{2}}X\left(:,j\right),\;\;\;\;\;\;\mathrm{if}\;\|X{\left(:,j\right)\|}_{2}>\frac{\lambda }{\mu^{\left(k\right)}}\\ 0,\;\;\;\;\;\;\;\;\;\;\;\;\;\;\;\;\;\;\;\;\;\;\;\;\;\;\;\;\;\;\mathrm{otherwise} \end{array}\right. \end{array}$$

where $X\left(:,j\right)$ represents the *j*-th column of the matrix X.

After $T_{\left(2\right)}^{k+1}$ is solved, it can be transformed into tensor form $T^{k+1}$.

(iii) Fix the rest of the variables and update of N is obtained by Equation (16):

(16) $$N^{k+1}\begin{array}{c} =\arg\underset{N}{\min}\left(\eta {\left\|N\right\|}_{F}^{2}+\frac{\mu^{k}}{2}\|D-B^{k+1}-T^{k+1}+\frac{Y^{k}}{\mu^{k}}-N{\|}_{F}^{2}\right) \end{array}$$

Differentiating it with respect to N, and let it be zero:

(17) $$2\eta N-\mu^{k}\left(D-B^{k+1}-T^{k+1}+\frac{Y^{k}}{\mu^{k}}-N\right)=0$$

Then, we have the following:

(18) $$N^{k+1}\begin{array}{c} =\frac{\mu^{k}}{2\eta +\mu^{k}}\left(D-B^{k+1}-T^{k+1}+\frac{Y^{k}}{\mu^{k}}\right) \end{array}$$

(iv) Fix the rest of the variables and update of Y:

(19) $$Y^{k+1}\begin{array}{c} =Y^{k}+\mu^{k}\left(D-B^{k+1}-T^{k+1}-N^{k+1}\right) \end{array}$$

(v) Update of μ:

(20) $$\mu^{\left(k+1\right)}=\min\left(\rho \mu^{\left(k\right)},\mu_{\max}\right)$$

where 0<ρ<1, $\mu_{\max}=10^{5}$.

(vi) Iteration termination condition:

(21) $$\frac{\|D-B^{k+1}-T^{k+1}-N^{k+1}{\|}_{F}}{{\left\|D\right\|}_{F}}<\varepsilon$$

where $10^{-3}<\varepsilon <10^{-5}$.

Finally, we summarize the algorithmic process of the proposed SLRTD method in Algorithm 2.

From target-background separation, the tensor D is decomposed into the defect target T and background B. The defect target image $f_{T}$ and background image $f_{B}$ are reconstructed from T and B, respectively. Finally, the defect targets can be extracted and segmented by the adaptive thresholding segmentation method.
**Algorithm 2:** Solving Equation (7) by ADMM**Input:** Original defect image sequence tensor
D∈R^m×n×l,
power p, λ,
η **Output: **
B,
T,
N
**Initialize: **D, $T^{\left(0\right)}=B^{\left(0\right)}=N^{\left(0\right)}=0$, W=I, Y=0, $\mu_{\max}=10^{5}$, ρ=1.1, k=0 **While: not converged**, **do** **step 1:** Update $B^{k+1}$ by Equation (11) **step 2:** Update $T^{k+1}$ by Equation (15) **step 3:** Update $N^{k+1}$ by Equation (16) **step 4:** Update $Y^{k+1}$ by Equation (19) **step 5:** Update $\mu^{\left(k+1\right)}$ by Equation (20) **step 6:** Check the convergence condition **step 7:** Update k=k+1 **end While**

### 3.3. Model Analysis

#### 3.3.1. Computational Complexity

For surface defect image *D*, it could be constructed into the tensor $D\in {\mathbb{R}}^{m\times n\times l}$ with sliding window’s size m×n and times l. The computation complexity of the solution of B includes three operations [34] of FFT, SVD, and GST; FFT operation is $O\left(Amnl\log\left(l\right)\right)$, SVD operation is $O\left(Amn^{2}\lceil \frac{l+1}{2}\rceil \right)$, GST operation is $O\left(Bmn\right)$, where A denotes iterations number in Algorithm 1, B denotes iterations number in GST. Thus, the computation complexity of solution B is $O\left(Amnl\log\left(l\right)+Amn^{2}\lceil \frac{l+1}{2}\rceil +Bmn\right)$. For solution T, the computation complexity is $O\left(mn\right)$. Therefore, the whole computation complexity of model is $O\left(C\left(Amnl\log\left(l\right)+Amn^{2}\lceil \frac{l+1}{2}\rceil +Bmn\right)+mn\right)$, where C is the iterations number in ADMM.

#### 3.3.2. Convergence of Algorithm

**Figure 4:**
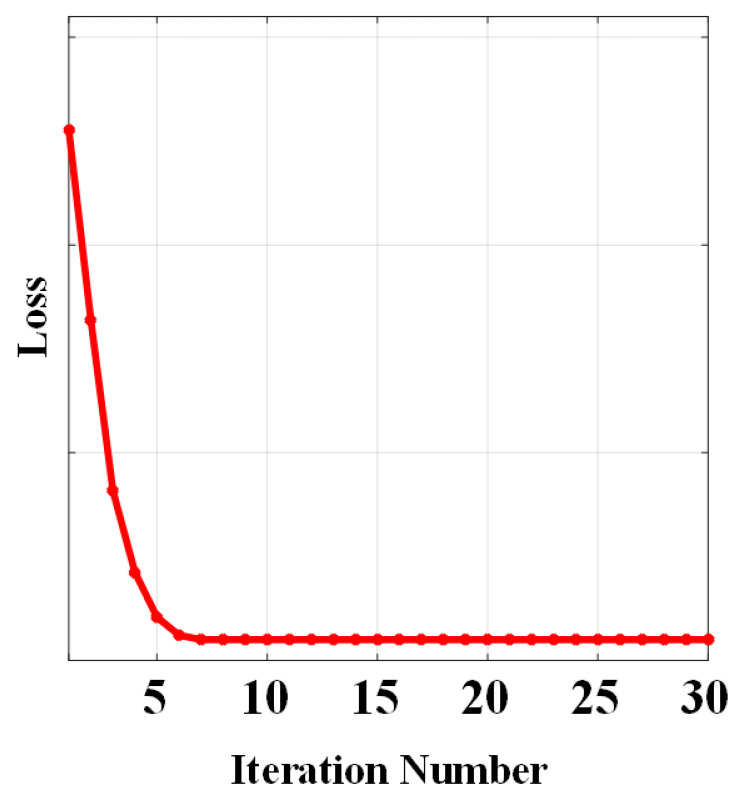
Convergence curve of the proposed SLRTD method.

The convergence of model Equation (9) has been proven in [25]. We evaluate the convergence of the proposed SLRTD method to empirically show the convergence through experiments in different iterations. We set a convergence error value of $\|D-B-T-N{\|}_{F}/{\left\|D\right\|}_{F}$ and found that the algorithm takes approximately 0.5 s per frame for small target detection. Figure 4 shows the variation in convergence error as the number of iterations progresses from 0 to 30. It can be observed that after 10 iterations, the error of our algorithm gradually decreases, and the convergence curve stabilizes, indicating that our method meets the convergence requirements and successfully achieves optimal or near-optimal solutions.

## 4. Experiment

To evaluate the effectiveness of the proposed SLRTD method, we conduct a series of qualitative and quantitative experiments. Firstly, the details of data collection and preprocessing, and evaluation metrics are described. Secondly, the key parameters and robustness are analyzed. Finally, the performance comparison is conducted between our method and the other four typical methods.

### 4.1. Experimental Setup

#### 4.1.1. Data Collection and Preprocessing

**Figure 5:**
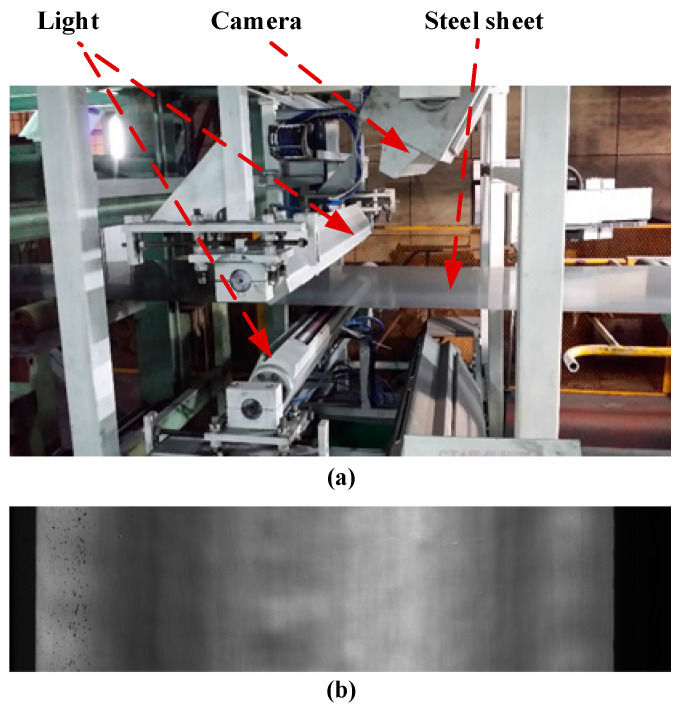
Industrial image acquisition platform in the galvanized strip steel production line: (**a**) strip steel production line; (**b**) image captured from the steel surface.

**Figure 6:**
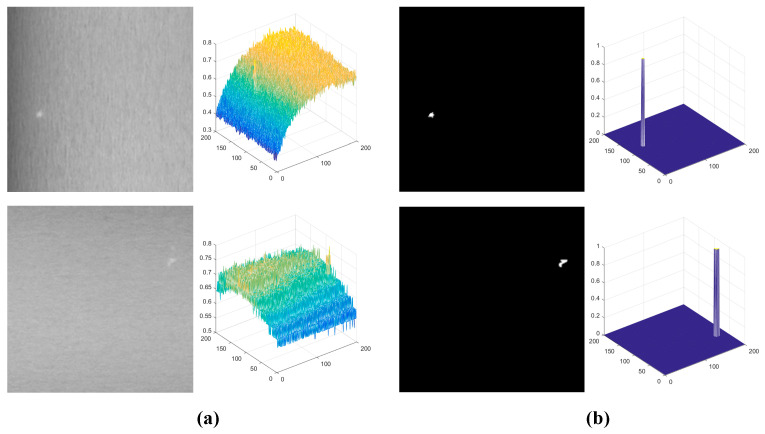
Three-dimensional maps of defect samples and the corresponding ground-truth: (**a**) original image; (**b**) ground-truth.

The proposed SLRTD method is validated on a dataset from a real-world galvanized strip steel production line, and the image acquisition platform can be observed in Figure 5. We crop the raw images’ 4096 × 1024 pixels into 200 × 200 pixels to make the dataset. In total, we obtain 100 defect images and 100 non-defect images. For each image, the pixel-level ground-truth is manually marked by using “1” to denote defective pixels and “0” to denote defect-free pixels. The size of the white-spot defect in the production line is about 1 mm to 3 mm, which is about 5 to 8 pixels in the 200 × 200 image. Defect samples and the corresponding masks of the constructed datasets are shown in Figure 6.

#### 4.1.2. Evaluation Metrics

In order to evaluate the detection performance of the proposed SLRTD model, Precision, Recall, Precision-Recall (P-R) curve, receiver operating characteristic (ROC) curve, area under ROC curve (AUC) average F-Measure (*F_ζ_*), and mean square error (MAE) are adopted as evaluation metrics.

Precision is used to evaluate how many positive pixels are correctly classified. It can be defined as follows:

(22) $$\mathrm{Precision}=\frac{TP}{TP+FP}$$

where true positive (TP) indicates the number of defect pixels which are correctly classified as defect, false positive (FP) indicates the number of background pixels mistakenly identified as defect.

Recall is used to evaluate how many pixels are correctly classified. It can be defined as follows:

(23) $$\mathrm{Recall}=\frac{TP}{TP+FN}$$

where false negative (FN) indicates the number of defect pixels that are not classified to defect class.

$F_{\zeta }$ represents the weighted harmonic mean of precision and recall. It can be defined as follows:

(24) $$F_{\zeta }=\frac{1}{N}\overset{N}{\underset{i=1}{\sum }}\frac{\left(\zeta^{2}+1\right)\times \mathrm{Precision}\times \mathrm{Recall}}{\zeta^{2}\times \mathrm{Precision}+\mathrm{Recall}}$$

MAE measures the dissimilarity between segmentation images BW and corresponding ground-truth image G. It can be defined as follows:

(25) $$\mathrm{MAE}=\frac{\sum_{i=1}^{H}\sum_{j=1}^{W}\left|BW\left(i,j\right)-G\left(i,j\right)\right|}{H\times W}$$

where H and W denote height and width of surface defect image.

Using Otsu’s threshold, we determine the Precision and Recall by changing the thresholds from 0 to 255 to obtain pairs of Precision and Recall.

### 4.2. Validation of the Proposed SLRTD Method

#### 4.2.1. Parameter Analysis

For the proposed SLRTD method, the patch size, sliding step size, and value of Schatten-p are evaluated by the AUC and MAE, where the regularized parameter λ is set to $\lambda =\frac{l}{\sqrt{\max\left(m,n\right)}}$, which is adaptive to the patch size and number of patches.

- Patch size

To balance the detection performance and computational complexity, we change the patch size from 20 to 50 with 10 intervals and provide the corresponding AUC and MAE in Table 1. As the patch size increases, the detection performance of the SLRTD method decreases. When the patch size is 50 × 50, the degradation is more serious. The reason is the larger patch size may destroy the low-rank and sparsity between the different patches, and influence the decomposition of the defect target and background.

b. Step size

In practice, we hope to take a larger step in exchange for the reduction in computational complexity. We fix the patch size and vary the step size from 10 to 40 with 10 intervals. The evaluation results are shown in Table 1. It could be observed that the performances of larger steps are always better than that of smaller ones, but when the step size is similar with the patch size, the performance becomes worse.

c. Value of Schatten-p

As shown in Table 2, the AUC and MAE with p change from 0.1 to 1 with 0.1 intervals. The detection performance of p=1 is worse than lower p as the over-shrinkage is serious. When p is decreased, the low-rank background may be closer to true rank, while more high-rank components would become zeros [34]. When p is too small, it would also make some low-rank backgrounds become zeros to lead the bad detection performance. Therefore, in order to make the tradeoff between detection accuracy and error, p is set to 0.7 in the following experiments.

#### 4.2.2. Robustness to Noise

**Figure 7:**
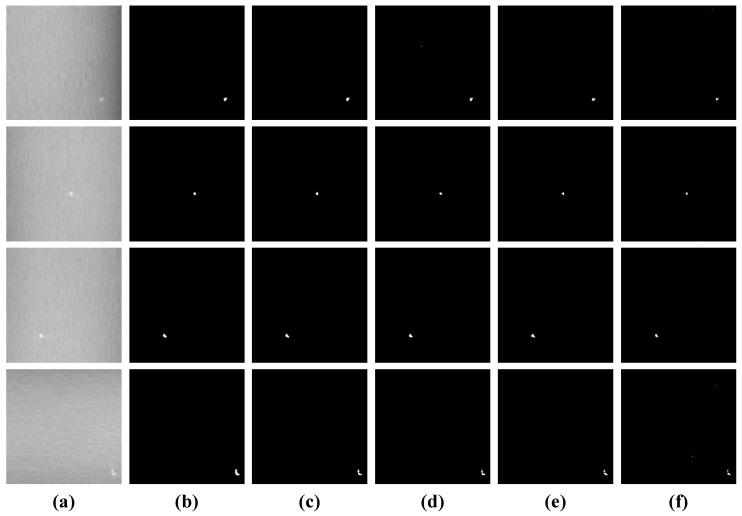
Detection results of binarization by Otsu’s method in different noise cases: (**a**) original image; (**b**) ground-truth; (**c**) 0 dB; (**d**) 36 dB; (**e**) 32 dB; (**f**) 28 dB.

The noise conditions of a real-world galvanized strip steel production line are complex and random; we evaluate the proposed SLRTD method’s performance in the noise case with different levels. Additive Gaussian noise with different signal-to-noise ratios (SNRs) are introduced to the original defect image, including 36 dB, 32 dB, and 28 dB. The experimental results are shown in Figure 7 and Table 3. It can be observed that the SLRTD is robust to noise in most cases; binarization defect images obtained by SLRTD are clear and accurate. When SNR decreases gradually, the AUC and MAE metrics can remain a relatively high level; for example, AUC still remains around 0.85 under the condition of SNR=28 dB, which is considered as less sensitive to noise. However, some pixels of the background are misclassified for defects, which is still a great challenge for the SLRTD method.

### 4.3. Comparison with the State-of-the-Art Methods

The proposed SLRTD method is compared with four state-of-the-art algorithms, including TRPCA [23], PSTNN [24], ETRPCA [25], and NN-TRPCA [29].

#### 4.3.1. Qualitative Comparison

**Figure 8:**
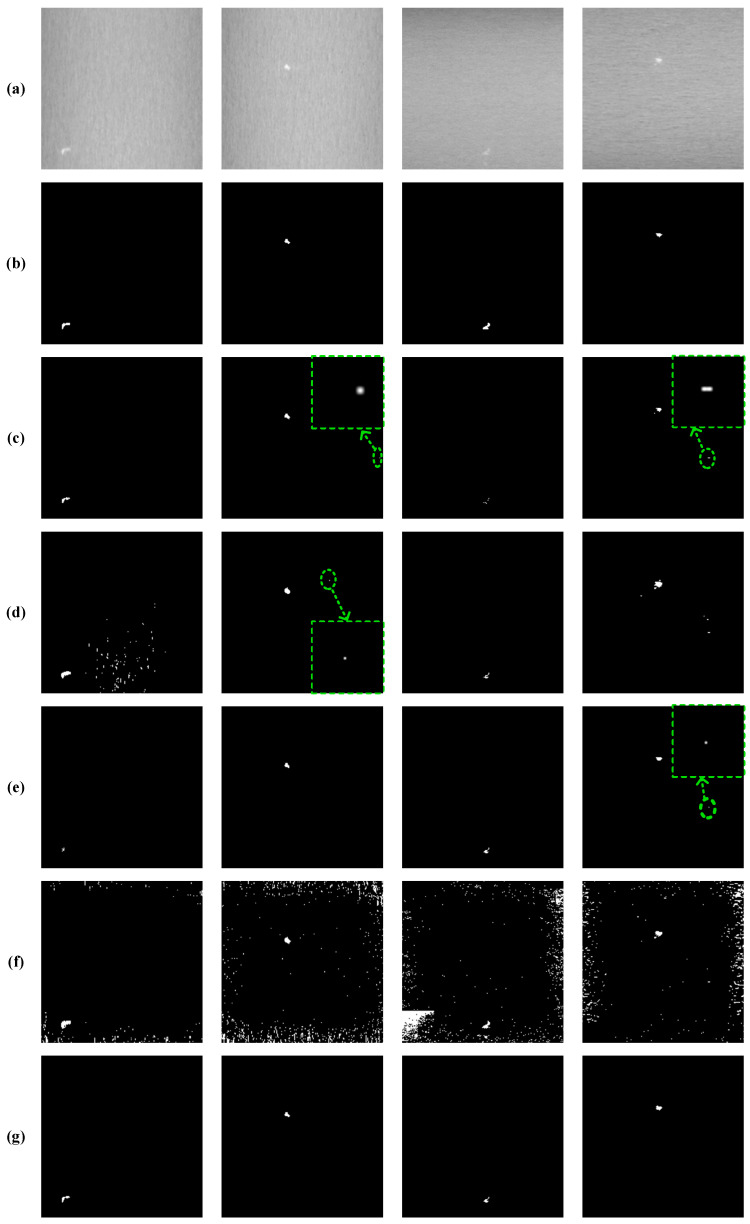
Qualitative comparison results: (**a**) input image; (**b**) ground-truth image; (**c**) TRPCA; (**d**) ETRPCA; (**e**) NN-TRPCA; (**f**) PSTNN; (**g**) ours.

To facilitate a more intuitive comparison of the performance of each algorithm, we selected representative defect images from the dataset. The false-detected targets are highlighted with green circles, which are shown in Figure 8. The qualitative comparison results between the proposed SLRTD method and the other four methods are shown in Figure 8. These images show various illumination and gray levels. NN-TRPCA and PSTNN can enhance defect targets, but they also enhance many clutters and noises of the background and the defect object cannot be uniformly highlighted, which can cause a high false alarm rate. These methods may lose the targets with heavy noise. NN-TRPCA exhibits poor robustness in detecting small targets against complex backgrounds, resulting in considerable background residue. The TRPCA and ETRPCA methods can detect the targets in the 2nd and 4th rows, but some background residue still remains. There are many pixels that belong to the background and are misjudged by defects, which leads to low accuracy. By contrast, SLRTD separates the defect objects from the image background successfully and locates various defects precisely. It more efficiently highlights the whole defect object with well-defined boundaries than the other methods. It can be concluded that WSNM regularization could improve the performance. In addition, WSNM regularization of SLRTD contributes to good performance, and thus, is the important factor to obtain more precise segmentation results than other methods. What is more, it is reasonable to conclude that treating the matrix singular values differently is the method by which the most important characteristics of the defects or background can be preserved. The superiority of the proposed SLRTD method can suppress the noise and detect the defect targets clearly, which can generate high-quality binary segmentation results by a simple threshold method.

#### 4.3.2. Quantitative Comparison

**Figure 9:**
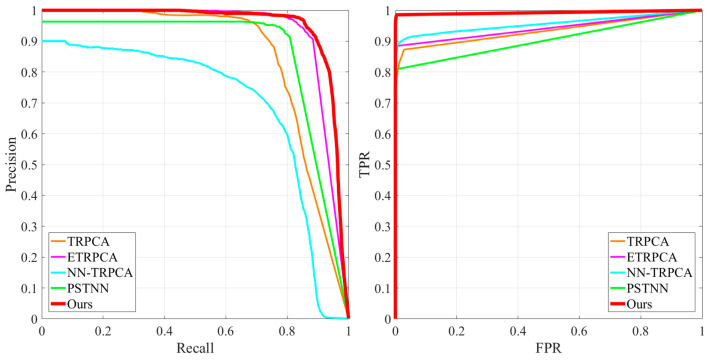
Quantitative comparison results with P-R curves and ROC curves.

To further demonstrate the superiority of the proposed SLRTD method, four competitive methods of TRPCA, ETRPCA, NN-TRPCA, PSTNN are selected for comparison. From the P-R and ROC curves demonstrated in Figure 9, for the same false alarm ratio, our method can achieve the highest detection performance. By imposing a p-norm to the patch-image, SLRTD can suppress background noise effectively for all defect images and lay a good foundation for the subsequent target segmentation. Table 4 summarizes the quantitative results of five methods, and the best results are marked in bold. It demonstrates that SLRTD also has better performance than the other four methods. Most of the AUC results are higher than 85%, and SLRTD achieves 0.9560. MAE of SLRTD is typically the lowest among all the methods. Compared with TRPCA, it is increased by 2.08% and 1.55% in AUC and MAE, respectively. Based on the above qualitative and quantitative analyses, it confirms that our proposed SLRTD method consistently outperforms some state-of-the-art methods and verifies the effectiveness of the proposed SLRTD method.

## 5. Conclusions

In order to detect small white-spot surface defects of galvanized strip steel accurately and rapidly, the SLRTD method is proposed in this paper. The nonlinear reweighting strategy based on Schatten p-norm is adopted to separate the defect image into a smooth non-defect background and random noise. A solution framework is proposed by the ADMM algorithm to obtain the defect target image. Based on the self-constructed defect dataset, experiments are conducted by the qualitative and quantitative method, which achieves the best performance among the state-of-the-art defect detection methods. In the future, we will focus on improving the ability of the proposed SLRTD method to detect the other tiny defects under weak illumination conditions or irregular defect-like texture surfaces. In the future, we will conduct binocular and structured-light stereo vision to obtain three-dimensional information of surface defects in industrial production.

## Figures and Tables

**Table 1:** Experimental results of AUC and MAE with different patch sizes and step sizes.

Patch	Step	AUC	MAE
20 × 20	10	0.9341	0.0009
	20	0.9712	0.0030
30 × 30	10	0.9501	0.0007
	20	0.9728	0.0019
	30	0.9775	0.0046
40 × 40	10	0.9560	0.0005
	20	0.9737	0.0017
	30	0.9769	0.0034
	40	0.9762	0.0034
50 × 50	10	0.9589	0.0006
	20	0.9732	0.0014
	30	0.9760	0.0021
	40	0.9744	0.0018
	50	0.9731	0.0015

**Table 2:** Experiment of AUC and MAE with different p.

*p*	AUC	MAE
0.1	0.9994	0.0613
0.2	0.9895	0.0263
0.3	0.9887	0.0105
0.4	0.9769	0.0042
0.5	0.9737	0.0018
0.6	0.9658	0.0009
0.7	0.9560	0.0005
0.8	0.9415	0.0005
0.9	0.9326	0.0005
1	0.9255	0.0005

**Table 3:** Experiment with different noise levels.

	SNR	No Noise	36 dB	32 dB	28 dB
Index					
AUC		0.9560	0.9386	0.9058	0.8272
MAE		0.005	0.1610	0.1731	0.1939

**Table 4:** Comparison of AUC and MAE of different methods.

	Method	TRPCA	ETRPCA	NN-TRPCA	PSTNN	Ours
Index						
AUC		0.9352	0.9259	0.9427	0.8925	0.9560
$F_{\zeta }$		0.5407	0.6569	0.6730	0.4028	0.8364
MAE		0.0160	0.0004	0.0071	0.0003	0.0005

## Data Availability

The data are not publicly available due to privacy issues.
